# Bending Stiffness of Honeycomb Paperboard

**DOI:** 10.3390/ma16010156

**Published:** 2022-12-24

**Authors:** Gabriela Kmita-Fudalej, Włodzimierz Szewczyk, Zbigniew Kołakowski

**Affiliations:** 1 Centre of Papermaking and Printing, Lodz University of Technology, 93-005 Lodz, Poland; 2 Department of Strength of Materials, Lodz University of Technology, 90-537 Lodz, Poland

**Keywords:** honeycomb paperboard, bending stiffness, local buckling, elastic deformation, mechanical properties

## Abstract

This article analyzes the influence of the initial deflection of the flat layers on the bending stiffness (*BS*) of honeycomb paperboards and presents two methods for its calculation. Both methods allow for the determination of *BS* in the main directions in the plane of the paperboard, i.e., the machine direction (*MD*) and the cross direction (*CD*). In addition, they have been verified by comparing the calculation results with the results of the *BS* measurements. The first method allowed for the calculation of the *BS* of cellular paperboard based on the mechanical properties of the paper used for its production. The second method allowed for the estimation of the *BS* of cellular paperboard based on the bending stiffness of other honeycomb paperboards with the same raw material composition and the same core cell size but with different thicknesses. In the first analytical method for the calculation of the bending stiffness of cellular paperboard, which does not include the deflections of the flat layers, the calculation results significantly differ from the measurement results, and they are overestimated. The second of the presented *BS* calculation methods allowed for a much more accurate assessment of paperboard’s bending stiffness depending on its thickness.

## 1. Introduction

The first reference to the production of paper honeycomb cores was in 1901 [1]. Since then, the range of applications of products with honeycomb cores has been continuously expanding. Materials in the form of paperboard and laminates consisting of various types of boards with cores placed between them are used by many industries. They are used for primary and secondary packaging, inserts, pallets, furniture, door fillings, and partition walls, as well as for elements used in the aviation and automotive industries [2,3,4,5]. Products with honeycomb cores are popular because of their advantages, for example:

- High resistance to crushing and bending stiffness in relation to their specific weight [6,7];
- Good energy absorption, thermal, and acoustic insulation properties [8];
- Low cost of production via eco-friendly, renewable materials.

These properties are similar to those provided by laminates in the form of corrugated board, which are made of the same raw materials and behave similarly under load, both in laboratory tests and in practical applications. This allows researchers to use methods obtained in the tests of corrugated board for the evaluation of cellular paperboard.

In the literature, one can find many experimental studies on composite panels, such as those described in [9,10,11,12,13,14,15,16,17,18]. Theoretical work on the analysis of the stability of composite structures has also been presented in the literature [19,20,21,22]. However, these studies do not concern the analysis of a board’s strength. Currently, numerical methods, such as those employed in references [23,24,25,26,27,28], are most often used to analyze the strength properties of board packaging. Numerical methods are also often used for the strength analysis of paperboard, particularly with respect to corrugated board [29,30,31,32]. Occasionally these methods are used to analyze specific issues, as in [33,34,35], in which the results of tests on creased paperboard are described. The authors of the above-mentioned publications investigated various properties, focusing not only on deformations and stresses occurring in loaded paperboard, but also examining the absorbed energy, such as in the research conducted by Bai et al. [31]. In these authors’ research, various measurement techniques are used, which are often complicated and require special equipment, such as Allansson et al. [29], who used a system of cameras tracking the displacement of markers in the form of spots on the paperboard’s surface to determine paperboard deformations. In [36,37], Hua et al. described the results of research on corrugated and cellular boards. Many different types of tests are also carried out in the case of cellular paperboard and laminates containing honeycomb cores. In [38], Chen and Yan conducted tests on laminates consisting of fiberboards and a honeycomb core. In [39], Wang tested honeycomb cores’ ability to absorb dynamic loads. A similar topic was dealt with by Gu et al. in [40], who determined the energy absorption of honeycomb paperboard subjected to compressive loads. In references [41,42,43,44,45], the finite element method was used to describe the behavior of cellular paperboard subjected to flat crushing.

In order to obtain average engineering properties of cellular and porous materials, the methods of homogenization [46,47], tolerance averaging, asymptotic-tolerance, and asymptotic-consistent methods are used.

The use of complex measurement and calculation techniques require non-standard equipment that are not used in industrial plants; therefore, they are difficult to apply in industrial practice.

Real structures, i.e., structures with imperfections, have inevitable geometrical inaccuracies, residual stresses, and deviations in shape, material properties, workmanship, etc. Theoretical considerations most often include imperfections of the geometrical type, i.e., initial deflections. These deflections often integrate all types of imperfections, i.e., all imperfections are reduced to a substitute initial deflection.

This article provides easy-to-use methods for predicting bending stiffness that are based on the commonly used physical properties of fibrous raw materials used in the production of cellular boards. In an analytical model based on Classical Laminate Theory (CLT) [48] and basic formulas from the field of the strength of materials, without accounting for the deflection of flat layers, the degree to which this imperfection of the honeycomb paperboard has an impact on the bending stiffness was proven.

The second of the presented *BS* calculation methods, based on the measurements of three bending stiffness values of paperboards, accounts for the influence of product imperfections and local buckling on the calculation value. This approach makes it possible to predict the *BS* of a given type of paperboard from three paperboards of different thicknesses. It can be used in honeycomb paperboard plants to predict the *BS* of paperboards. In addition, the phenomenon of the local buckling of the flat layers has generally been ignored in the research conducted so far.

According to the authors, this is the novelty of the proposed method.

## 2. Materials and Methods

**Figure 1:**
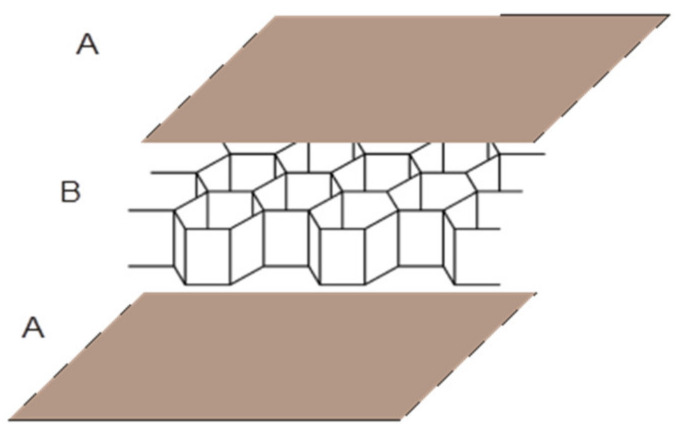
Construction of honeycomb paperboard [43].

The object of analysis was honeycomb paperboard. Honeycomb paperboard consists of two outer layers, A, and the honeycomb core, B, as illustrated in Figure 1.

**Figure 2:**
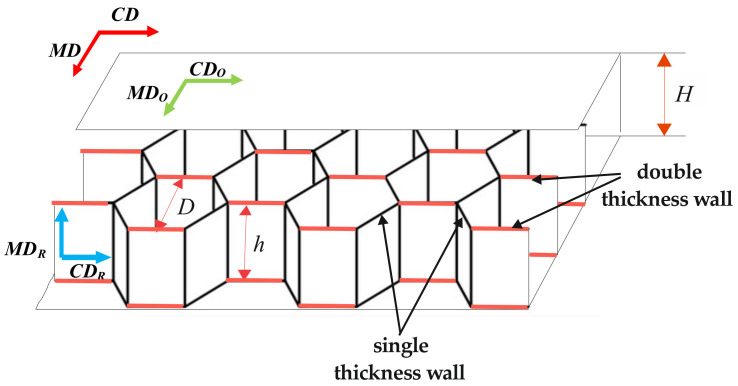
Construction of a cellular paperboard with a honeycomb core [43].

In the plane of cellular paperboard, two main directions of orthotropy can be considered. The first one covers the direction of manufacturing (called the machine direction *MD*). The second (perpendicular) direction is called the cross direction (*CD*). The main directions of the *CD* and *MD* of the paperboard are the same as the paper used for the flat layers (*CD_O_* and *MD_O_*—Figure 2). In the case of a paperboard core, the machine direction of the paper applied for the *MD_R_* core is parallel to the height of the core. However, the cross direction *CD_R_* is perpendicular to the height of the core.

The geometrical parameters of the cellular paperboard are described as: *D* —diameter of the circle drawn in the regular hexagon (defined as the cell size), *h*—core height, and *H*—paperboard thickness. Single-thickness walls have the thickness of the paper applied to manufacturing the cellular paperboard’s core. However, walls glued to each other have double thickness.

### 2.1. Experimental Research

**Figure 3:**
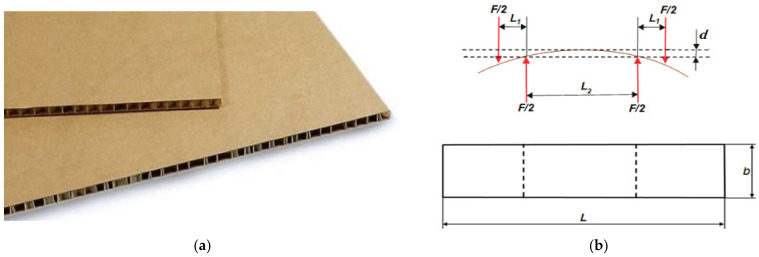
View of paperboard panel component (**a**) and scheme of support with dimensions of sample (**b**).

The experimental study was conducted at the Center of Papermaking and Printing (Lodz University of Technology). Measurement of paperboard’s bending stiffness was carried out using a 4-point bending process corresponding to the scheme depicted in Figure 3b and according to the standard PN-EN 5628:1995 [49]. The object of analysis was honeycomb paperboard. Such paperboards are produced in the form of the panels presented in Figure 3a. For the tests, rectangular samples were cut from a sheet of honeycomb paperboard. According to Figure 3b, the dimensions of sample are as follows: the total length *L* = 500 mm and width *b* = 100 mm. The distance between the supports and applied forces amounted to $L_{2}=200$ mm and $2L_{1}+L_{2}=400$ mm, respectively. Due to the orthotropic properties of honeycomb paperboard, the measurements were carried out in two main directions (*MD* and *CD*).

The bending stiffness measurement was calculated using Equation (1) based on dimensions from Figure 3b and is expressed in Nm, corresponding to the standard PN-EN 5628:1995 [49].

(1) $$BS=\frac{F\;L_{1}L_{2}^{2}}{16db}\;\;\left[\mathrm{Nm}\right]$$

where
*F*—acting force, N;
*L*_1_, *L*_2_—distances between supports, m;
*d*—deflection, m;
*b*—sample width, m.

**Figure 4:**
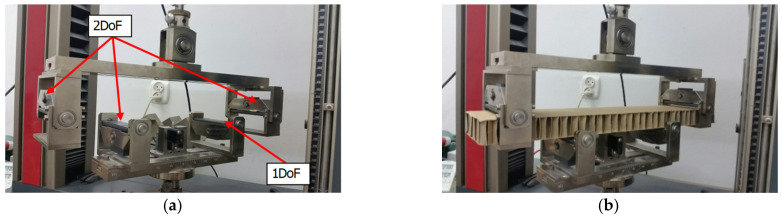
Tool for measuring bending stiffness (**a**) and a sample of the honeycomb board placed in the tool (**b**) [43].

Before performing tests of bending, samples were dried at temperature of 40 °C; subsequently, they were conditioned according to standard PN-EN 20187:2000 (i.e., at temperature of 23 ± 1 °C and relative air humidity of 50 ± 2%) [50]. This bending test was carried out on Zwick Tensile Machine model Z010 from the Zwick Roell Group (Ulm, Germany), equipped with specialized tools (Figure 4a). The load range of machine is from 0.1 N to 10 kN. The tool consists of four supports. Three of them (two upper supports and one lower) have two DoF and the fourth one has only one DoF. Supports with 2 DoF have the ability to rotate about two axes parallel to the principal directions in the plane of the cellular paperboard, and the support with 1 DoF can only rotate about an axis perpendicular to the plane in which the bending moment acts. During tests, supports moved with the velocity of 10 mm/min. The method of placing the sample in the measuring grip is shown in Figure 4b. The result of the determination in each direction is given as the average value obtained after testing ten samples.

Then, measurements of the physical properties of the materials used for the individual layers of the tested cellular paperboards were carried out. The subjects of the research are honeycomb paperboards with different raw material compositions and geometric parameters. The characteristics of the honeycomb paperboards and the physical characteristics of the paper from which they were made are presented in Table 1 and Table 2.

The TL200/FL140/TL200 paperboard had cores with a regular cell shape, similar to a regular hexagon, and the cells of the TL135/TL135/TL135 paperboard cores had irregular shapes in the form of hexagons with different side lengths. Different types of cellular paperboard, having the same raw material composition and different core heights and sizes, were produced at different times. For this reason, the fibrous raw material supplied for the production of individual paperboard could differ in terms of its physical properties from the paper samples used in laboratory tests to determine the physical properties of individual paperboard’s component layers.

### 2.2. The Method for Calculation BS of the Honeycomb Paperboard Based on the Mechanical Properties of Raw Materials (Method I)

The analytical model is based on the Classical Laminate Theory (CLT) [48] and basic formulas from the strength of materials. According to the CLT, all quantities characterizing the paperboard are reduced to the middle surface of the paperboard. In this article, in order to simplify the formulas, the influence of the coupling matrix (B) on the values of longitudinal and flexural stiffnesses [48] for flat layers has been omitted.

The middle layer of the paperboard, i.e., the honeycomb core, was considered as a series of bent beams. In the case of paperboard’s bending stiffness in the machine direction of *BS_MD_*, two single-thickness walls of the core cell were taken into account, while in *BS_CD_* one double-thickness wall of the core cell was accounted for.

In the bending test of cellular paperboard, assuming that no local buckling will occur in any of its layers, which may appear in the first phase at low loads and deflections, the bending stiffness of cellular paperboard, *BS*, can be calculated as the sum of the bending stiffness of the individual layers:

(2) $$BS’=\overset{3}{\underset{i=1}{\sum }}BS_{i}$$

where
*i*—layer number;
*BS_i_*—bending stiffness of the *i*^th^ layer in relation to the neutral axis of the paperboard.

In papermaking, the bending stiffness is related to the bending width of the sample *b*; thus, Equation (2) takes the form:

(3) $$BS=\overset{3}{\underset{i=1}{\sum }}\frac{BS_{i}}{b}$$

The bending stiffness in the machine direction *BS_MD_* of the cellular paperboard can be calculated:

(4) $$BS_{MD}=\frac{1}{b}\left(\frac{E_{MD1}\cdot J_{MD1}}{1-\nu_{MDCD1}\cdot \nu_{CDMD1}}+E_{MD2}\cdot J_{MD2}+\frac{E_{MD3}\cdot J_{MD3}}{1-\nu_{MDCD3}\cdot \nu_{CDMD3}}\right)$$

where
*E_MDi_*—Young’s modulus of the *i*^th^ layer in the machine direction of the honeycomb paperboard;
*ν_MDCDi_*, *ν_CDMDi_*—Poisson’s ratio of the *i*^th^ layer;
*J_MDi_*—moment of inertia of the section perpendicular to the *MD* of the *i*^th^ layer with respect to the neutral axis of the paperboard’s bending section.

Similarly, the bending stiffness in the cross direction of the cellular paperboard can be calculated as follows:

(5) $$BS_{CD}=\frac{1}{b}\left(\frac{E_{CD1}\cdot J_{CD1}}{1-\nu_{MDCD1}\cdot \nu_{CDMD1}}+E_{CD2}\cdot J_{CD2}+\frac{E_{CD3}\cdot J_{CD3}}{1-\nu_{MDCD3}\cdot \nu_{CDMD3}}\right)$$

where
*E_CDi_*—Young’s modulus of *i*^th^ layer in the cross direction of the paperboard;
*J_CDi_*—moment of inertia of the section perpendicular to the *CD* of the *i*^th^ layer in relation to the neutral axis of the bending section of the paperboard.

The Poisson ratios of paper are rarely determined, but—based on the results presented by Baum [51]—it can be assumed that the product *ν_MDCD_∙ν_CDMD_* has approximately the same value for various types of paper with similar structure. For paper and solid board used for the production of corrugated and cellular paperboard, the average value of the products was 0.14, and this value was used for the calculations.

After taking the above assumption into account, Equations (4) and (5) take the form of Equations (6) and (7), respectively:

(6) $$BS_{MD}=\frac{1}{b}\left(\frac{E_{MD1}\cdot J_{MD1}}{0.86}+E_{MD2}\cdot J_{MD2}+\frac{E_{MD3}\cdot J_{MD3}}{0.86}\right)$$

(7) $$BS_{CD}=\frac{1}{b}\left(\frac{E_{CD1}\cdot J_{CD1}}{0.86}+E_{CD2}\cdot J_{CD2}+\frac{E_{CD3}\cdot J_{CD3}}{0.86}\right)$$

The presented relations allow for the calculation of the bending stiffness of the cellular paperboard based on known geometrical parameters and the Young’s modulus of its component layers.

**Figure 5:**
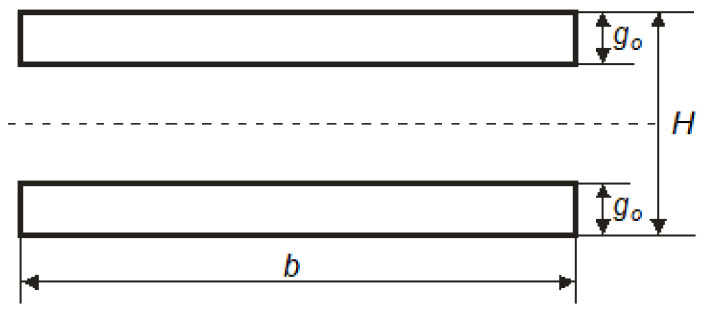
Diagram of a simplified honeycomb paperboard cross-section.

Cellular paperboard most often has both flat layers made of the same material. Considering such a case involving paperboard with a symmetrical structure, the total moment of inertia of the flat layers, *J_O_*, can be calculated from Equation (8), for which the cross-section is illustrated in Figure 5

(8) $$J_{o}=\frac{b\left(H^{3}-{\left(H-2g_{o}\right)}^{3}\right)}{12}$$

where
*H*—honeycomb paperboard’s thickness;
*b*—sample width;
*g_o_*—thickness of paperboard’s flat layer.

**Figure 6:**
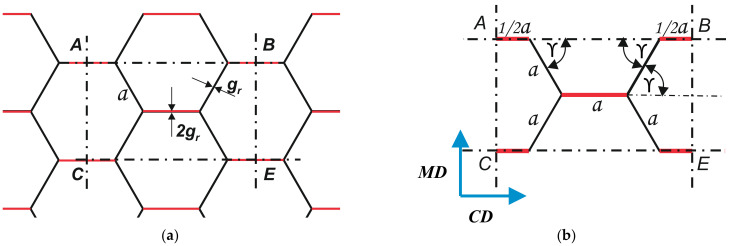
Periodic cells: *ABCE*—periodic cell separated from the paperboard core (**a**); dimensions of the periodic core cell (**b**).

Inserting (8) into (6) and (7) yields the approximate values of *BS_MD_* and *BS_CD_*. In order to develop more accurate calculation models, the stiffness of a repeating element of the cellular paperboard structure was considered. A periodic cell *ABCE* was isolated from the honeycomb core structure (Figure 6a). It was assumed that the cross-section of the core cell with a plane parallel to the flat layer has the shape of a regular hexagon with side *a* and diameter *D* (diameter of the circle inscribed in the hexagon), between which there is the following relationship: a=D√3.

The dimensions of a periodic cell in the shape of a regular hexagon can be determined via the following relationship:

(9) CE=AB=2·a+2·a ·cosγ=3 a

(10) $$AC=BE=2\cdot a\;\cdot \sin\gamma =a\sqrt{3}\;$$

The total moment of inertia of the flat layers in the machine direction, *Jo_MD,_* and in the cross direction, *Jo_CD_*, of the periodic cell under consideration was calculated from Equations (11) and (12) while substituting the appropriate cell size for *b*:

(11) $$J_{oMD}=\frac{3a\left(H^{3}-{\left(H-2g_{o}\right)}^{3}\right)}{12}$$

(12) $$J_{oCD}=\frac{a\sqrt{3}\left(H^{3}-{\left(H-2g_{o}\right)}^{3}\right)}{12}$$

The moments of inertia of the cross-section in the cross direction of the core section with a width in the cross direction of 3a, which corresponds to the dimension of the repeating cell, was determined from the following relationship:

(13) $$J_{MD2}=J_{rMD}=\frac{g_{r}}{3\surd 3}\cdot {\left(H-2g_{o}\right)}^{3}$$

where *g_r_*—thickness of the paper from which the core is made.

The moments of inertia of the section in the machine direction of the core with a width in the machine direction of a√3, corresponding to the dimension of the repeating cell, were determined from the following relationship:

(14) $$J_{CD2}=J_{rCD}=\frac{1}{6}g_{r}\cdot {\left(H-2g_{o}\right)}^{3}$$

In the case of cellular paperboard with a symmetrical structure, the Young’s modulus of the flat layers are the same and can be described as follows: *E_MD_*_1_ = *E_MD_*_3_ = *E_OMD_*, while *E_CD_*_1_ = *E_CD_*_3_ = *E*_O*CD*_. The Young’s modulus of the core, *E*_2_, depending on the orientation of the core walls in the plane of the paperboard, assumes different values, but never exceeds the value of the Young’s modulus of the paper used for the production of the core in the cross direction of the paper *E_rCD_.* Due to the inability to determine the exact values of the Young’s modulus of the core in the machine directions and cross directions of the cellular paperboard, their values were assumed to be equal to *E_rCD_*. The consequence of this assumption is the overestimation of the core bending stiffness with respect to the bending stiffness of the entire cellular paperboard.

Considering the assumptions in (6) and (7) concerning the Young’s modulus and the widths of bent samples corresponding to the dimensions of the repeating cell, i.e., the width of the sample bending in the machine direction *b* = 3*a*, and the sample bending in the cross direction *b* = $a\sqrt{3}$, we obtain the following:

(15) $$BS_{MD}=\frac{1}{3\;a}\left(\frac{E_{oMD}\cdot J_{oMD}}{0.86}+E_{rCD}\cdot J_{rMD}\right)$$

(16) $$\;BS_{CD}=\frac{1}{\sqrt{3}\;a}\left(\frac{E_{oCD}\cdot J_{oCD}}{0.86}+E_{rCD}\cdot J_{rCD}\right)$$

where
*E_oCD_*—Young’s modulus of paper used to produce flat layers of cellular paperboard in cross direction;
*E_oMD_*—Young’s modulus of paper used to produce flat layers of cellular paperboard in machine direction;
*E_rCD_*—Young’s modulus of paper used to produce core of paperboard in cross direction.

**Figure 7:**
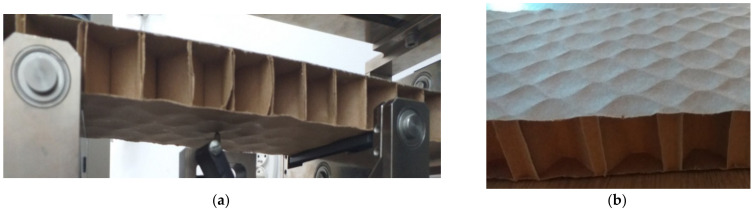
Buckling of compressed flat surface of cellular paperboard (**a**), defects in the shape of the flat layer of honeycomb paperboard (**b**).

Equations (15) and (16) make it possible to determine the bending stiffness in the case when, during the bending test, the phenomenon of buckling in the component layers does not occur, or then it has a negligible effect on the relationship between the load causing the sample to bend and its deflection arrow. Local buckling may occur when the paperboard is bent in the compression layer (Figure 7a).

The force-deflection diagram of the cellular paperboard in which the flat layer buckles is approximately linear. Moreover, the deformations are reversible, that is, they can be considered elastic. However, the compression of the flat layer, due to the local deflections in the area of individual core cells, will result in the lower bending stiffness of the paperboard compared to the case in which there are no local deflections of the flat layer.

Another factor influencing the shape of the board’s bending curve is the initial shape of the cover surfaces. Figure 7b shows the undulations of the covering glued with respect to the core of the cellular paperboard, which has not been subjected to loads that can be described as the so-called initial deflections.

In both the outer layer subject to stretching and the compressed layer, the preliminary bends cause larger deformations in the plane of the material when the paperboard is loaded compared to the deformations of flat plates.

The bending stiffness of cellular paperboard is influenced, among other things, by the shape of the core cell. In practice, there are cores that differ significantly from the regular type. An example is the paperboard shown in Figure 7b. In this case, the core cross-section does not have the shape of a regular hexagon. Single-thickness walls are much longer than double thickness walls. This is due to the insufficient width of the adhesive joints applied to the component layers of the core during its gluing.

Cellular paperboard is made of paper that can originate from a variety of sources. Despite the use of the same types of paper, even from one supplier, this difference in origin causes differences in both the structural–dimensional and strength properties of the raw material. This has a significant impact on the strength properties of the finished product and thus on the accuracy of *BS* calculations.

### 2.3. The Method of Calculating the BS of Cellular Paperboard Based on the Results of Bending Tests (Method II)

There is a method that allows one to easily calculate the bending stiffness of paperboard, which is based on a simplification ignoring the influence of the core on the bending stiffness of paperboard described in [52]. The dependence of the bending stiffness (*BS*) on the paperboard thickness (*H*) can be described by a second-order polynomial

(17) $$BS=b\prime H^{2}+cH$$

where *b*′, *c*—constants.

Applying such a simplification to cellular paperboard, with its high thickness, may cause large discrepancies between the actual and calculated values.

In order to eliminate the influence of factors causing *BS* calculation errors, such as the irregularity of the cores or initial local deflections of the flat surfaces, a new method was developed that accounts for the influence of all layers on the value of paperboard’s bending stiffness. Substituting (11) and (13) into (15) and (12), as well as (14) into (16) after transforming the formulas in both cases, both in the machine and cross directions, we obtain the dependence of the bending stiffness *BSα* on the paperboard thickness *H* described by the polynomial third degree:

(18) $$BS_{\propto }=a_{\alpha }H^{3}+b_{\alpha }H^{2}+c_{\alpha }H+d_{\alpha }$$

where
*α* = *MD* or *CD*;
*a_α_*, *b_α_*, *c_α_*, *d_α_*—constants described by the properties of the material used for the flat layers.

Applying such a simplification to cellular paperboard, with its high thickness, may cause large discrepancies between the actual and calculated values. Since for *H* = 0, bending stiffness *BS* = 0, parameter *d_α_* is equal to 0.

Knowing the thickness and bending stiffness of at least three cellular paperboards of a given type, i.e., with flat layers made of the same material and having the same core cell with the same segments, the parameters *a_α_*, *b_α_*, and *c_α_* can be determined. This allows for the bending stiffness of this type of board of any thickness in the *MD* and *CD* to be estimated by the Equation (18).

## 3. Results and Discussion

**Figure 8:**
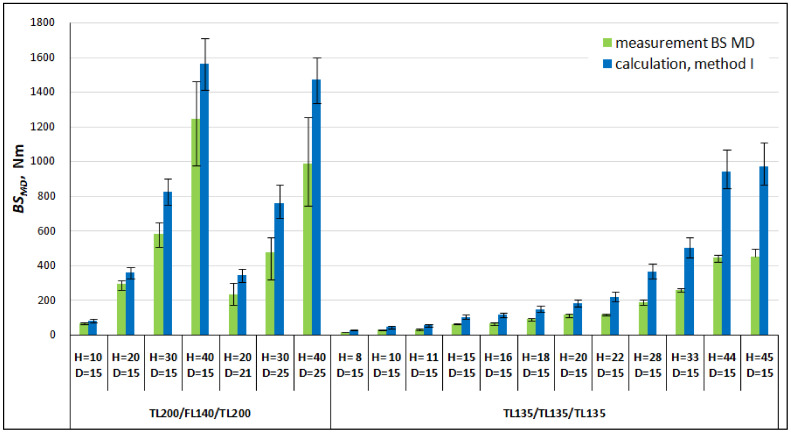
Comparison of the results and calculations of bending stiffness in the machine direction (*MD*).

**Figure 9:**
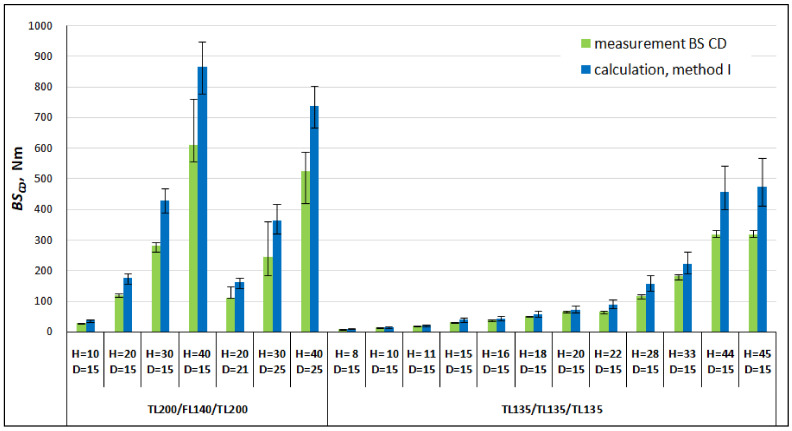
Comparison of the results and calculations of bending stiffness in the cross direction (*CD*).

In order to validate the first calculation method, Table 3 and Figure 8 and Figure 9 compare the results of the bending stiffness calculations made with the use of Equations (15) and (16) with the results of the measurements made in the machine direction *MD* and cross direction *CD*. The calculation error in this study is defined as the absolute value of the difference between the measurement and calculation result, which is divided by the measurement value and expressed as a percentage.

Large discrepancies between the results of the measurements and calculations were found. In all cases, the calculated *BS* values for both the *MD* and *CD* of the honeycomb paperboards were significantly higher than the measured ones.

The tests showed that the core stiffness in paperboard increases with an increasing paperboard thickness and decreases with an increasing segment core cell size. In the examined cases, despite the fact that the value of the core’s bending stiffness was overestimated by overestimating the Young’s modulus, *E_rCD_*, its share with respect to the paperboard stiffness in the machine direction was small and varied from approx. 3% to approx. 15% of the paperboard stiffness, but in the cross direction it was up to 45% in the case of the thick paperboard.

**Figure 10:**
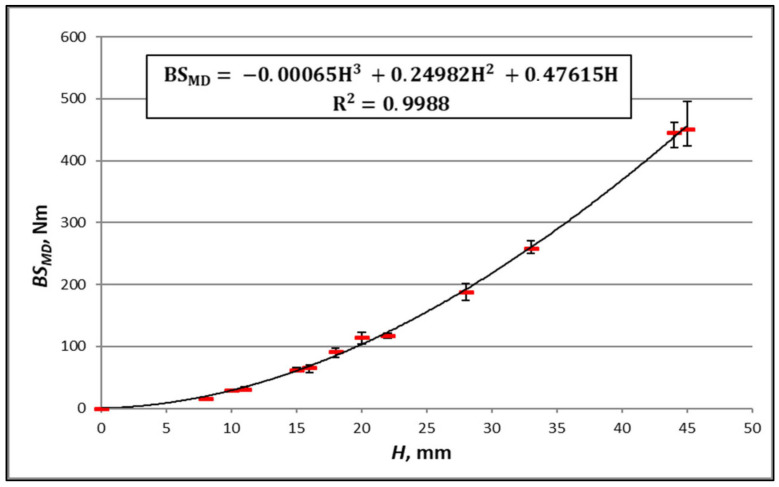
Measurements of *BS_MD_* boards TL135/TL135/TL135 with core cell size of *D* = 15 mm and different thicknesses.

**Figure 11:**
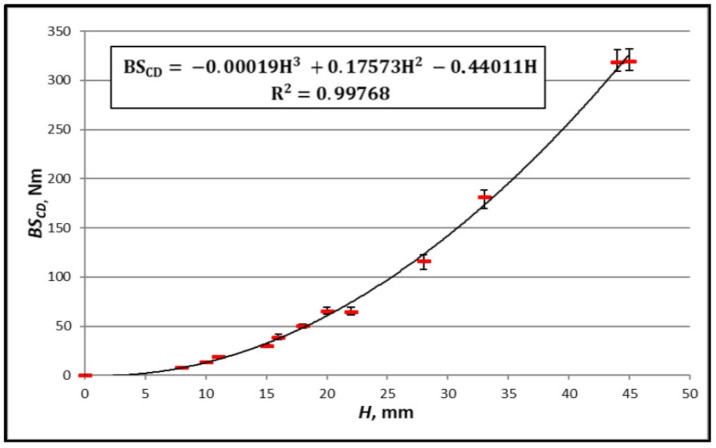
Measurements of *BS_CD_* boards TL135/TL135/TL135 with core cell size of *D* = 15 mm and different thicknesses.

**Figure 12:**
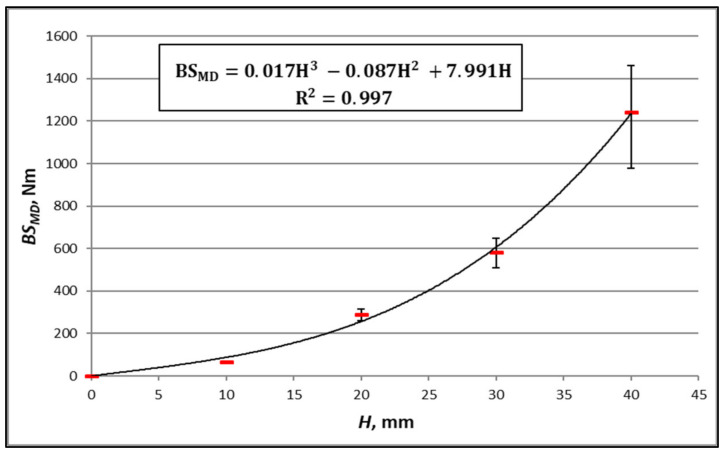
Measurements of *BS_MD_* boards TL200/FL140/TL200 with core cell size of *D* = 15 mm and different thicknesses.

**Figure 13:**
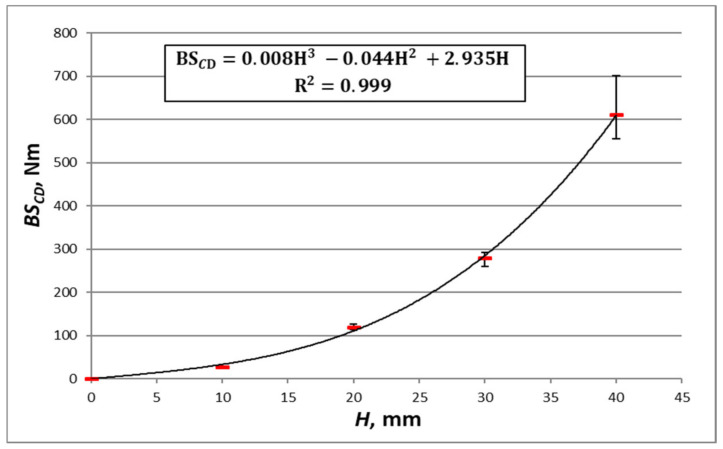
Measurements of *BS_CD_* boards TL200/FL140/TL200 with core cell size of *D* = 15 mm and different thicknesses.

In order to ascertain the performance of the second method, Figure 10, Figure 11, Figure 12 and Figure 13 show the results of the measurements of the bending stiffness of the paperboards with the same material composition, depending on their thickness.

Figure 10, Figure 11, Figure 12 and Figure 13 show trend lines based on all the measured points. As can be seen, the variability of paperboard’s *BS* as a function of its thickness *H* can be described with great accuracy using a third-degree polynomial (Equation (18)). The absolute values of the differences between the measurement and calculation are in all cases small, but relative to the measured values, they are in some cases large. An example is the case of the paperboard with a thickness of 10 mm, as shown in Figure 12. The absolute difference between the measured and calculated value is similar to that of the paperboard with thicknesses of 20 mm and 40 mm, but the relative difference related to the measured value is high and amounts to 36% of the measured value.

As it is clear to see, a problem occurs in the case of the thin paperboard with low bending stiffness. To avoid this problem, a rule has been adopted that the method of determining bending stiffness will be applied to the paperboard whose thickness is within the range determined by the smallest and the greatest thicknesses of the paperboard used to determine the relationship between *BS* and *H*. To ensure the accuracy of the calculations in the entire range of paperboard thicknesses, particularly larger thicknesses, it was assumed that one of the paperboards—based on the measurement results, for which the parameters of Equation (18) were determined—was the paperboard with the highest thickness.

Another problem related to the determination of the relationship between *BS* and *H* is the difference in the thickness of the cellular paperboards used for this purpose. When the minimum number of measurement points (paperboard) necessary to determine the *a_α_*, *b_α_*, and *c_α_* constants is used, it may be the case that even small measurement errors with slight differences in paperboard thickness will cause a large error in determining the *a_α_*, *b_α_*, and *c_α_* constants and, as a result, a significant discrepancy between the results of the measurements and calculations. Such a case is illustrated by the measuring points in Figure 10 and Figure 11, which were determined based on *BS* measurements of the paperboard with thicknesses of 44 mm and 45 mm. In order to prevent this type of error, the rule was adopted that the difference in thickness between the honeycomb paperboards used to determine the *a_α_*, *b_α_*, and *c_α_* constants must not be less than 11% of the tested thickness range.

Table 4 and Table 5 present the exemplary results of the *BS_MD_* and *BS_CD_* calculations made using Equation (18) and the error values that have been rounded to integers. The calculation error in this study is defined as the absolute value of the difference between the measurement and calculation result, which is divided by the measurement value and expressed as a percentage.

The simplified method of calculating the *BS* of cellular paperboard described by Equations (15) and (16) yielded results with a greater error in the tested cases than the mathematical model described by Equation (18). Table 6 shows the maximum and average errors of the *BS* calculations made with the use of Equations (15), (16) and (18); the error values were rounded to integers.

## 4. Conclusions

In the case of the analytical approach to the cellular board bending test, for Equations (15) and (16), when the deflections of the flat layers are not accounted for, the calculation results significantly differ from the measurement results, and they are overestimated—in two cases by more than 100% of the measured value. However, this method makes it possible to evaluate the contribution of the core’s bending stiffness to the stiffness of the entire cellular paperboard. The tests have shown that this share increases with the height of the core (paperboard thickness) and decreases with the increase in the core’s cell size. In the analyzed cases, the influence of the core with respect to the bending stiffness of the cellular paperboard in the machine direction reached a maximum of 15%, and this value was 45% in the cross direction of the *BS* of the entire paperboard.

The second of the presented *BS* calculation methods, based on the measurements of three values of bending stiffness, allows for a much more accurate assessment of paperboard’s bending stiffness depending on its thickness. This method includes the influence of product imperfections on the values of the calculations. It mainly concerns imperfections such as the lack of flatness of the layers or the inaccuracy of the shape of the core cells.

None of the analyzed methods can eliminate the influence of the non-uniformity of the fibrous material on the value of the *BS* calculations, but the use of the Equation (18) allowed us to limit the maximum calculation error to 27% of the measured value and the mean value of calculation errors to 10%.

## Figures and Tables

**Table 1:** Characteristics of the tested honeycomb paperboards.

Designation	D [mm]	H1 (Given by the Manufacturer) [mm]	H (Measured) [mm]	Raw Material of Flat Layers	Weight [g/m^2^]	Core Raw Material	Weight [g/m^2^]
**TL200/FL140/TL200**	15	10	9.87	Testliner	200	Fluting	140
	15	20	19.85				
	15	30	29.50				
	15	40	39.80				
	21	20	19.69				
	25	30	28.81				
	25	40	39.64				
**TL135/TL135/TL135**	15	8	8.07	Testliner	135	Testliner	135
	15	10	10.19				
	15	11	11.31				
	15	15	15.37				
	15	16	16.22				
	15	18	18.27				
	15	20	20.20				
	15	22	22.03				
	15	28	28.05				
	15	33	32.62				
	15	44	43.87				
	15	45	44.54				

**Table 2:** Physical characteristics of the paper.

Designation	Material	Weight [g/m^2^]	Paper Thickness [mm]	Young’s Modulus of Paper in Machine Direction [GPa]	Young’s Modulus of Paper in Cross Direction [GPa]
**TL200**	Testliner	200	0.264	5.70	2.37
**FL140**	Fluting	140	0.203	5.50	2.18
**TL135**	Testliner	135	0.204	3.55	1.11

**Table 3:** Results of *BS* measurements and calculations using method I and calculation error.

Designation	D [mm]	H [mm]	Measurement *BS_MD_* [Nm]	Calculation *BS_MD_* [Nm]	Calculation Error *BS_MD_* [%]	Calculation *BS_MD_* Core [Nm]	Measurement *BS_CD_* [Nm]	Calculation *BS_CD_* [Nm]	Calculation Error *BS_CD_* [%]	Calculation *BS_CD_* Core [Nm]
**TL200/FL140/TL200**	15	9.87	64.97	83.40	28	2.68	28.35	37.58	33	4.02
	15	19.85	290.88	359.02	23	23.66	119.21	174.93	47	35.49
	15	29.50	583.81	826.61	42	79.72	280.11	430.12	54	119.57
	15	39.80	1241.35	1564.07	26	198.48	610.52	865.50	42	297.72
	21	19.69	232.02	346.32	49	16.48	112.00	161.86	45	24.72
	25	28.81	475.22	756.69	59	44.50	245.42	362.87	48	66.76
	25	39.64	986.81	1472.09	49	117.63	525.81	739.60	41	176.44
**TL135/TL135/TL135**	15	8.07	15.95	26.85	68	0.75	8.08	9.26	15	1.13
	15	10.19	28.61	43.62	52	1.57	13.90	15.45	11	2.35
	15	11.31	30.36	54.21	79	2.17	19.05	19.47	2	3.25
	15	15.37	62.37	102.68	65	5.61	30.41	38.65	27	8.42
	15	16.22	65.17	114.78	76	6.61	38.90	43.62	12	9.92
	15	18.27	91.18	147.22	61	9.54	50.24	57.20	14	14.31
	15	20.20	114.29	181.58	59	12.97	65.69	71.98	10	19.46
	15	22.03	117.88	217.84	85	16.92	64.93	87.96	35	25.38
	15	28.05	187.97	362.49	93	35.36	116.35	154.94	33	53.04
	15	32.62	257.95	499.11	93	55.94	181.21	221.95	22	83.90
	15	43.87	445.89	942.05	111	137.49	318.92	456.86	43	206.24
	15	44.54	450.94	973.10	116	143.89	319.30	474.14	48	215.84

**Table 4:** Exemplary results of *BS_MD_* and *BS_CD_* calculations using method II and calculation errors for honeycomb paperboard TL135/TL135/TL135 and *D* = 15 mm.

*H* (mm) of the Paperboard from Which the *BS* Was Calculated		*H* [mm]											
		8	10	11	15	16	18	20	22	28	33	44	45
**8, 15, 45**	***BS_MD_*/*BS_CD_* [Nm]**	16/8	26/13	33/16	62/30	71/35	90/45	110/56	132/68	207/114	275/163	437/304	451/319
	**Calculation error** ***BS_MD_*/*BS_CD_* [%]**	0/0	8/7	7/17	0/0	9/10	1/11	3/15	12/5	10/2	7/10	2/5	0/0
**10, 18, 45**	***BS_MD_*/*BS_CD_* [Nm]**	-	29/14	35/17	64/34	73/39	91/50	111/63	133/76	206/125	274/174	436/306	451/319
	**Calculation error** ***BS_MD_*/*BS_CD_* [%]**	-	0/0	14/9	3/12	12/1	0/0	3/5	13/18	9/7	6/4	2/4	0/0
**11, 33, 45**	***BS_MD_*/*BS_CD_* [Nm]**	-	-	30/19	57/37	64/43	81/55	100/68	120/82	190/133	258/181	433/307	451/320
	**Calculation error** ***BS_MD_*/*BS_CD_* [%]**	-	-	0/0	9/23	1/10	11/9	13/4	2/27	1/14	0/0	3/4	0/0
**15, 20, 45**	***BS_MD_*/*BS_CD_* [Nm]**	-	-	-	62/30	72/37	92/50	114/66	138/82	216/139	286/191	439/309	451/319
	**Calculation error** ***BS_MD_*/*BS_CD_* [%]**	-	-	-	0/0	10/6	1/0	0/0	17/27	15/19	11/5	2/3	0/0
**28, 33, 45**	***BS_MD_*/*BS_CD_* [Nm]**	-	-	-	-	-	-	-	-	188/116	258/181	434/310	451/320
	**Calculation error** ***BS_MD_*/*BS_CD_* [%]**	-	-	-	-	-	-	-	-	0/0	0/0	3/3	0/0

**Table 5:** Results of *BS_MD_* and *BS_CD_* calculations using method II and calculation errors for honeycomb paperboard TL200/FL140/TL200 and *D* = 15 mm.

*H* [mm] of the Paperboard from Which the *BS* Was Calculated		*H* [mm]			
		10	20	30	40
**10, 20, 40**	***BS_MD_*/*BS_CD_* [Nm]**	65/28	291/118	682/301	1241/605
	**Calculation error *BS_MD_*/*BS_CD_* [%]**	0/0	0/1	17/7	0/1
**10, 30, 40**	***BS_MD_*/*BS_CD_* [Nm]**	65/27	226/97	584/256	1241/553
	**Calculation error *BS_MD_*/*BS_CD_* [%]**	0/3	22/19	0/9	0/9
**20, 30, 40**	***BS_MD_*/*BS_CD_* [Nm]**	-	291/114	584/261	1241/564
	**Calculation error *BS_MD_*/*BS_CD_* [%]**	-	0/5	0/7	0/8

**Table 6:** Calculation errors, *BS*.

Designation of the Formula Used for the Calculation	Board Designation	Direction in Plane of the Board	Maximum Calculation Error [%]	Average Calculation Error [%]
**Equations (15) and (16)**	TL135/TL135/TL135	*MD*	116	80
		*CD*	48	23
	TL200/FL140/TL200	*MD*	59	39
		*CD*	54	44
**Equation (18)**	TL135/TL135/TL135	*MD*	17	7
		*CD*	27	9
	TL200/FL140/TL200	*MD*	22	6
		*CD*	19	10
The equation in Figure 10	TL135/TL135/TL135	*MD*	22	6
The equation in Figure 11	TL135/TL135/TL135	*CD*	15	7
The equation in Figure 12	TL200/FL140/TL200	*MD*	36	14
The equation in Figure 13	TL200/FL140/TL200	*CD*	16	11

## Data Availability

Not applicable.
